# Multiple Stochastic Parameters Influence Genome Dynamics in a Heterozygous Diploid Eukaryotic Model

**DOI:** 10.3390/jof8070650

**Published:** 2022-06-21

**Authors:** Timea Marton, Christophe d’Enfert, Melanie Legrand

**Affiliations:** Institut Pasteur, Université Paris Cité, INRAE USC2019, Unité Biologie et Pathogénicité Fongiques, F-75015 Paris, France; timea.marton@pasteur.fr (T.M.); christophe.denfert@pasteur.fr (C.d.)

**Keywords:** *Candida albicans*, loss-of-heterozygosity, heterogeneity, genome instability

## Abstract

The heterozygous diploid genome of *Candida albicans* displays frequent genomic rearrangements, in particular loss-of-heterozygosity (LOH) events, which can be seen on all eight chromosomes and affect both laboratory and clinical strains. LOHs, which are often the consequence of DNA damage repair, can be observed upon stresses reminiscent of the host environment, and result in homozygous regions of various sizes depending on the molecular mechanisms at their origins. Recent studies have shed light on the biological importance of these frequent and ubiquitous LOH events in *C. albicans*. In diploid *Saccharomyces cerevisiae*, LOH facilitates the passage of recessive beneficial mutations through Haldane’s sieve, allowing rapid evolutionary adaptation. This also appears to be true in *C. albicans*, where the full potential of an adaptive mutation is often only observed upon LOH, as illustrated in the case of antifungal resistance and niche adaptation. To understand the genome-wide dynamics of LOH events in *C. albicans*, we constructed a collection of 15 strains, each one carrying a LOH reporter system on a different chromosome arm. This system involves the insertion of two fluorescent marker genes in a neutral genomic region on both homologs, allowing spontaneous LOH events to be detected by monitoring the loss of one of the fluorescent markers using flow cytometry. Using this collection, we observed significant LOH frequency differences between genomic loci in standard laboratory growth conditions; however, we further demonstrated that comparable heterogeneity was also observed for a given genomic locus between independent strains. Additionally, upon exposure to stress, three outcomes could be observed in *C. albicans*, where individual strains displayed increases, decreases, or no effect of stress in terms of LOH frequency. Our results argue against a general stress response triggering overall genome instability. Indeed, we showed that the heterogeneity of LOH frequency in *C. albicans* is present at various levels, inter-strain, intra-strain, and inter-chromosomes, suggesting that LOH events may occur stochastically within a cell, though the genetic background potentially impacts genome stability in terms of LOH throughout the genome in both basal and stress conditions. This heterogeneity in terms of genome stability may serve as an important adaptive strategy for the predominantly clonal human opportunistic pathogen *C. albicans,* by quickly generating a wide spectrum of genetic variation combinations potentially permitting subsistence in a rapidly evolving environment.

## 1. Introduction

The genome of the human opportunistic fungal pathogen *C. albicans* is highly tolerant to genomic rearrangements, in particular loss of heterozygosity (LOH) events. This type of genomic rearrangement is ubiquitously found across sequenced chromosomes of both clinical and laboratory strains. LOH events are often the result of DNA double-strand breaks (DSBs) as these DNA damages are preferentially repaired through homologous recombination (HR)-mediated repair mechanisms in *C. albicans* [1,2,3]. The LOH size is often dictated by the type of HR mechanism used, with homozygosis that can be limited to a given locus, or span across a chromosomal arm or the entire chromosome [4]. By increasing the number of DNA breaks, using nucleases or exposure to DNA damaging agents mimicking stress, the frequency of LOH events has been shown to increase in *C. albicans* [1,3,5]. Additionally, genomic instability resulting from perturbations of the yeast DNA repair machinery also augments the occurrence of LOHs [2,6,7,8,9,10]. Interestingly, Forche et al. have shown that various in vitro stress conditions increase the frequency of LOHs and influence the molecular mechanisms behind LOH occurrence [5]. 

Certainly, LOH plays an important role in the biology of *C. albicans* as (i) they have been shown to occur in vivo, both during murine intravenous infections [11] and in gastrointestinal [12] and oropharyngeal [13] colonization model and (ii) they are required at the mating-type-like (*MTL*) locus to make sterile *MTL* heterozygous cells into mating-competent *MTL*a/a or *MTL*α/α homozygous cells. In terms of their role in adaptation, LOHs have been associated to adaptive phenotypes such as antifungal resistance [14] and increased or reduced virulence [15,16]. In diploid organisms, LOH may contribute to rapid evolutionary adaptation by revealing recessive beneficial alleles [17,18]. On the other hand, these genomic rearrangement events may also unveil recessive deleterious or lethal alleles [1,19]. Therefore, within a predominantly clonal population, such genomic rearrangements contribute significantly to the purging of detrimental alleles and to the acquisition of genetic variation, thus rendering the understanding of LOH events more interesting.

In *C. albicans*, LOH events have been mostly described as markers of genomic instability, having for consequences the potential to generate new phenotypic traits. For instance, the increased sensitivity to the alkylating agent methyl methanesulfonate (MMS) that characterizes some derivatives of the reference strain SC5314 has been explained by an LOH event on the right arm of chromosome 3 (Chr3R), encompassing the *MBP1* locus, which encodes a component of a transcription complex involved in regulating G1/S genes important for DNA repair [20]. Likewise, a growth defect when proteins are the only available nitrogen source was also associated to an LOH event comprising one of the *SAP2* alleles (secreted aspartic protease) on the left arm of ChrR [21]. More recently, due to the convenience of whole-genome sequencing, evolutionary studies have highlighted the frequent occurrence of small LOH events over short time scales [12]. Besides genome sequencing*,* two major tools are commonly used to monitor the occurrence of LOH events in *C. albicans*. The first one, known as the *GAL1*/*URA3* system, uses the native *C. albicans GAL1* gene (located on the left arm of Chr1), encoding for a galactokinase implicated in galactose metabolism and the commonly used *URA3* auxotrophic marker, encoding orotidine-5’-phosphate decarboxylase involved in the biosynthesis of the nucleoside uridine. An artificial heterozygous locus is generated at the native *GAL1* locus by the replacement of one of the *GAL1* alleles with the *URA3* gene. LOH events at this locus are monitored by growing cells on media containing 2-deoxygalactose (2-DG) or 5-fluoroorotic acid (5-FOA), for *GAL1* and *URA3* counter-selection, respectively [22,23]. With this system, the evaluation of bidirectional LOH events (allowing to monitor loss of both haplotypes) is restricted to the endogenous *GAL1* locus. Nevertheless, unidirectional LOH events (monitoring the loss of a single haplotype) can still be assessed at various loci throughout the genome by generating heterozygous *URA3* loci [5]. The second system is a fluorescence-based system, known as the BFP/GFP LOH reporter system, and is engineered by integrating an artificial fluorescent heterozygous locus, where one of the chromosome homologs possesses the BFP-encoding gene and the other the GFP-encoding gene. Heterozygous cells at the locus of interest are therefore bifluorescent and LOH occurring at this locus can easily be monitored by tracking the loss of either the GFP or BFP signals using flow cytometry. Unlike the *GAL1/URA3* system, there is no locus restriction associated to the use of this system and high-throughput screening can easily be implemented with this fluorescence-based system. The BFP/GFP LOH reporter system has already allowed LOH screens to be performed with the overexpression of mutant collections to identify key regulators of genome stability in *C. albicans* [8]. Overall, previous studies relying on both systems have estimated that LOHs in standard laboratory growth conditions occur at a frequency of 10^−4^ in *C. albicans* [1,5,8,19].

Few studies have been specifically aimed at better understanding LOH dynamics in *C. albicans.* In the study by Forche et al. 2011, the authors analyzed the appearance of unidirectional LOH events using a collection of heterozygous KO mutants of genes located in 18 distinct genomic loci [5]. For each locus, they replaced one of the two alleles with the *URA3* auxotrophic marker and recorded the loss of *URA3* through the appearance of 5-FOA^R^ colonies. These experiments concluded that LOH rates in standard laboratory conditions are comparable between genomic loci and that the rates of LOH show a medium correlation with the distance between the *URA3* marker and the centromere. However, these estimations are based on the loss of a single haplotype, while we now recognize that chromosome homozygosis biases for numerous chromosomes exist in *C. albicans* due to the presence of recessive lethal or deleterious alleles on certain homologs, where a given haplotype is preferentially lost [1,19]. Additionally, the authors showed that different stress conditions induce augmentations of LOH at five genomic loci and that the extent of the increase correlates with the intensity of the stress [5]. However, the coordination of LOH events is yet unclear as the overall dynamics of LOH in *C. albicans* has not been thoroughly investigated. Additionally, the mining of sequencing data from in vivo- and in vitro-evolved laboratory strains and clinical strains have highlighted that LOH break points are more frequently observed in the proximity of repeat regions, suggesting the presence of hotspots for LOH events throughout the genome [12,24,25]. Altogether, it is still unknown if certain genomic loci exhibit higher LOH frequencies and what their biological impact is on the biology of *C. albicans*, potentially favoring adaptation.

The phenotype associated with a given genetic variant is undoubtedly influenced by the genetic background of the strain. In *C. albicans*, various observable phenotypic differences have been reported across different genetic backgrounds regarding biofilm formation, growth rates, or antifungal resistance assays [26,27,28]. Recently, a study illustrated the importance of the genetic background in biofilm formation, as diverse *C. albicans* genetic backgrounds exhibit clear phenotypic differences upon the knock-out (KO) of key regulators of biofilm formation, indicating circuit diversification among members of this species [29]. Depending on the genetic background of a strain, a mutation may be favorable in one environment but neutral or detrimental in another. Additionally, phenotypic heterogeneity has also been described upon the growth of *C. albicans* in fluconazole, where population subsets display contrasting phenotypes within the same genetic background [30]. Altogether, genetic background-specific effects on phenotypic expression suggest a phenotypic continuum, which could complexify genotype–phenotype association studies [31]. In *C. albicans*, such heterogeneity in terms of the appearance of genomic plasticity events has not been extensively described. A study conducted by Gerstein and Berman suggested that genetic background also influences the appearance of aneuploidy events and impacts the level of heterogeneity in terms of genome size within a single and between multiple genetic backgrounds during evolution experiments upon exposure to fluconazole [32], highlighting variations in the potential for adaptability between genetic backgrounds. However, unlike the model yeast *Saccharomyces cerevisiae* [33], heterogeneity in terms of LOH frequency in *C. albicans* has not yet been investigated.

Here, we used *C. albicans* as a model organism to investigate heterogeneity in terms of genome stability in a eukaryotic diploid. We aimed to study LOH frequency across its genome in order to investigate genome wide LOH dynamics and assess heterogeneity in LOH frequencies in both standard laboratory growth conditions and upon stress exposure. Overall, we compared LOH frequencies at multiple genomic loci as well as between multiple independent strains to assess the extent of heterogeneity in terms of LOH occurrence. The extent of heterogeneity in LOH frequency that we observed between independently constructed strains complicated the comparison of LOH frequency at multiple loci across the genome of *C. albicans* and led us to rethink the way that genome instability has been addressed in the past, highlighting the fact that cautions should be taken when drawing conclusions from analysis done with single strains. In addition to heterogeneity in standard laboratory growth conditions, we also observed heterogeneity in LOH frequency in the presence of stress, both in terms of the nature of the response (no effect, augmentation, or diminution), and in terms of response intensity. Our study suggests that LOH events may occur stochastically within a cell, though the genetic background potentially impacts genome stability in terms of LOH throughout the genome in both basal and stress conditions.

## 2. Materials and Methods

### 2.1. Strains and Culturing Conditions

*C. albicans* strains described in the study are derived either from SN148 (His^−^ Arg^−^ Ura^−^ Leu^−^) or SN95 (His^−^ Arg^−^) [34]. Yeast cells were cultured on/in rich YPD medium (1% yeast extract, 2% peptone, and 2% dextrose). Synthetic defined (SD) (0.67% yeast nitrogen base without amino acids and 2% dextrose) and synthetic complete (SC) (0.67% yeast nitrogen base without amino acids, 2% dextrose, and 0.08% drop-out mix with all the essential amino-acids) media were used for selection. Solid media were obtained by adding 2% agar. All *C. albicans* strains are listed in Table S2.

### 2.2. Blue-Green Collection Strain Construction

The Blue-Green (BG) collection is composed of a series of barcoded strains, each one possessing the BFP/GFP LOH reporter system on a given chromosome, designed to assess LOH frequencies throughout the *C. albicans* genome. These strains were constructed as described in Marton et al. [35]. These strains (Table S2) were selected as they do not display any obvious gross chromosomal rearrangement (GCR).

### 2.3. Strain Validation

#### 2.3.1. Basic Phenotyping

All strains underwent basic phenotypic characterization, additionally to the verification of the proper integration of cassettes at targeted loci by junction PCR. Functionality of auxotrophic markers was evaluated by drop tests on SC medium with appropriate dropout amino acids, depending on tested marker. Overnight saturated cultures in YPD of selected strains were spotted on YPD, SC-His, SC-Arg, SC-Ura, and SC-Leu and placed at 30 °C for 24 h to observe presence and absence of growth. Furthermore, the functionality/intensity of both fluorescent proteins (BFP and GFP) was validated by flow cytometry and fluorescence microscopy. The colony morphology of all strains was also assessed on both solid YPD and SD media, at 30 °C. Doubling times were evaluated in YPD medium at 30 °C by measuring the optical density with TECAN Infinite.

#### 2.3.2. Assessing Functionality of the LOH Reporter System

Additionally, functionality of the BFP/GFP LOH reporter system was also evaluated by exposing strains to genotoxic stress, ethyl methanesulfonate (MMS), and heat shock, and measuring the increase in mono-fluorescent populations. Homogenous BFP/GFP heterozygous precultures were obtained in SC-His-Arg medium overnight at 30 °C. Sixty million cells of the preculture were inoculated in 3 mL of YPD and incubated for 3 h at 30 °C, to obtain cells in exponential growth phase. The latter culture was divided into two (2 × 1.5 mL) and cells were pelleted and resuspended in either YPD at room temperature (control) or, YPD + MMS or pre-heated YPD (stress condition). The MMS stress assay is conducted in volumes of 3 mL YPD (control) or YPD + 0.03% MMS (stress) and exposed for 30 min at 30 °C. Then, cells are pelleted, washed with fresh YPD, and are recovered in 3 mL of YPD incubated at 30 °C overnight. The heat shock assay is performed in 200 µL of YPD (control) or pre-heated YPD at 50 °C (stress), cells are exposed to 51 °C for 90 s. Both heat-shocked and control samples are placed on ice for 5 min before inoculation into a fresh 3 mL of YPD and left to recover at 30 °C overnight. Following the recovery time, 10^6^ cells of each sample is analyzed by flow cytometry, using a MACSQuant analyzer (Miltenyi Biotec, Bergisch Gladbach, Germany), where the BFP is detected with a 405-nm laser and 425- to 475-nm filters and the GFP is detected with a 488-nm laser and 500- to 550-nm filters. To determine the number of LOH affected cells, FACS profiles are analyzed using FlowJo V10.1 software (BD Life Sciences, Ashland, OR, USA).

#### 2.3.3. Identifying the Delimitations of the Mono-Fluorescent Populations on FACS Profiles in Different Genomic Loci

The appearance of LOH events was triggered by heat shock in various strains of the Blue-Green collection to facilitate the identification, characterization, and delimitation of the flow cytometry-sorted populations, particularly the mono-fluorescent populations. Heat-shocked cultures were filtered using BD Falcon™ Cell strainers (Corning, Boulogne-Billancourt, France) to remove large debris. Cells were diluted in 1× PBS at a final concentration of at least 20 × 10^6^ cells/mL and kept on ice. The MoFlo^®^ Astrios™ flow cytometer (Beckman Coulter, Villepinte, France), located at the cytometry platform of the Institut Pasteur, Paris, was used to analyze and sort the cells of interest. For each sorted gate, 400 cells were recovered in 400 μL of liquid YPD medium. Sorted cells were plated on three YPD agar plates and incubated at 30 °C for 48 h. Recovered CFUs were counted and a subset of CFUs (*n* = 16) from each population was characterized by spot assay and by FACS. Spot assays were conducted on YPD (control), SC-His (to test the presence of the *BFP-HIS*1 cassette) and SC-Arg (to test the presence of the *GFP-ARG*4 cassette) while fluorescence status was assessed by FACS (10^4^ events per sample). Overall, all major populations identifiable in the FACS profiles were characterized, permitting the identification and the delimitation of mono-fluorescent populations (mono-BFP and mono-GFP) and double fluorescent population (BFP/GFP).

### 2.4. Fluctuation Assay to Determine LOH Frequency

**Figure 1:**
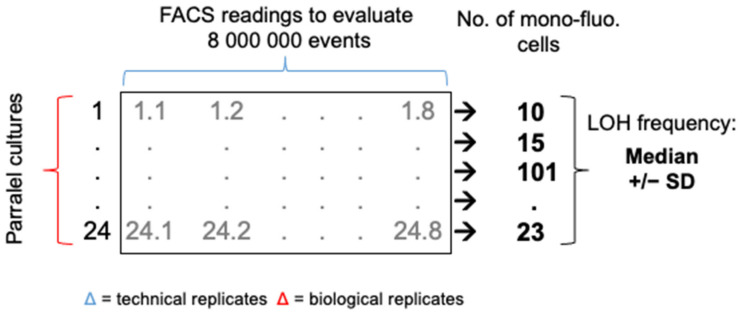
Schematic representation of the fluctuation analysis to determine an accurate LOH frequency. For a given strain in one growth condition, 24 parallel cultures are generated. For each parallel culture, 8 FACS analyses of 10^6^ cells are conducted for a total sampling size of 8 × 10^6^ cells. A total number of mono-fluorescent cells is calculated per parallel culture. The median LOH frequency (+/−SD) is assessed using the 24 parallel cultures.

LOH frequencies at different genomic loci and in different growth conditions were evaluated using the following fluctuation assay. A preculture was established by inoculating a single colony in liquid SC-His-Arg media and incubating overnight at 30 °C, assuring the starting population is homogenous at the BFP/GFP locus. One hundred cells of the preculture were then inoculated into multiple parallel cultures of 1 mL (YPD or YPD + stressor) in Deep-well 96 plates and incubated at 30 °C (or 39 °C) until stationary phase was reached (48 h growth and 72 h growth for fluconazole condition). For each parallel culture, 8 × 10^6^ cells were analyzed by FACS, 8 FACS analysis each assessing fluorescence of 10^6^ yeast cells, using the MACSQuant analyzer (Miltenyi Biotec, Bergisch Gladbach, Germany), where the BFP is detected with a 405-nm laser and 425- to 475-nm filters and the GFP is detected with a 488-nm laser and 500- to 550-nm filters. A total of 192 (24 parallel cultures) or 96 (12 parallel cultures) FACS output files per strain and condition were analyzed using the FlowJo V10.1 software. The gates to determine the LOH frequencies were selected according to the delimitations of the mono-fluorescent populations (as identified in the above section) conserved throughout sample analysis. Total number of mono-BFP and mono-GFP cells, from the 8 FACS profiles obtained per parallel culture, are added to determine a total number of cells that have undergone LOH. The absolute mono-fluorescence frequency for a given strain and condition represents the median (+/−SD) LOH frequency of 24 or 12 parallel cultures. Figure 1 is a schematic representation of the analysis matrix permitting to determine an LOH frequency for a strain in a condition.

### 2.5. Strain Construction for Assessing LOH Frequency Heterogeneity

Using the transient CRISPR-cas9 transformation protocol [36], both homologues were simultaneously targeted for transformation cassette integration. Thus, by directing a DNA DSB with a sgRNA, the BFP/GFP LOH reporter system can be engineered in only one transformation round. The BFP/GFP LOH reporter system was integrated in the most telomere proximal intergeneic region of ≥5 kb on Chr2L, Chr2R, or Chr5L, at the same loci selected in Marton et al. using the same sgRNA and repair templates [35]. The sgRNA and Cas9 cassettes utilized were conducted as described in Min et al. [36], from the pV1093 plasmid. While repair template cassettes were constructed using 120 bp primers, composed of 20 bp complementary to both the P*_TDH3_*-*GFP*-*ARG4* and P*_TDH3_*-*BFP*-*HIS1* cassettes and 100 bp tails possessing the complementary sequences of the targeted integration locus (Table S2). Each primer pair was utilized to amplify both the P*_TDH3_*-*GFP*-*ARG4* and P*_TDH3_*-*BFP*-*HIS1* cassettes from plasmid pCRBluntII-P*_TDH3_*-*GFP*-*ARG4* and plasmid pCRBluntII-P*_TDH3_*-*BFP*-Cd*HIS1*, respectively. Each cassette was amplified in a total PCR volume of 500 μL, precipitated in 100% ethanol and re-suspended in 100 μL of distilled sterile water. The SN95 parental strain, possessing both arginine and histidine auxotrophies, was co-transformed with 3 μg of P*_TDH3_*-*GFP*-*ARG4* cassette, 3 μg of P*_TDH3_*-*BFP*-*HIS1* cassettes, 1 μg of Cas9 cassette, and 1 μg of sgRNA using the lithium acetate/PEG transformation protocol. The transformants were selected on SD medium and junction PCRs were performed to ensure proper integration of both cassettes at the targeted locus. Additionally, these strains underwent basic phenotyping and the functionality of the LOH reporter system was validated as described in above sections.

### 2.6. Stress Conditions

Stress conditions and concentrations were selected based on literature and laboratory routines used to induce genomic instability; oxidative stress H_2_O_2_ 4 mM, fluconazole 10 µg/mL, and heat (39 °C). The fitness of each strain was evaluated by doubling time, in YPD medium with stressor at 30 °C by measuring the optical density with TECAN Infinite.

### 2.7. Two Reporter System Strain

A strain possessing two different LOH reporter systems, BFP/GFP (Chr5L) and *GAL1/URA3* (Chr1L) systems, was constructed to evaluate LOH frequencies in two distinct genomic loci simultaneously. The CEC5838 strain (Table S3) (Ura^−^ Leu^−^), possessing the BFP/GFP LOH reporter system on the left arm of Chr5, was transformed using the *URA3* knockout cassette targeting the *GAL1* locus on the left arm of Chr1. Repair template was constructed by PCR amplification from plasmid pFA-*URA3*, as described for the integration of the BFP/GFP LOH reporter, using primers in Table S3 and standard lithium acetate/PEG transformation protocol [37]. The transformants were selected on SD + leu medium, junction PCRs were performed to ensure proper integration of both cassettes at the targeted locus and strains underwent basic phenotyping (as described in above sections). The resulting strain is heterozygous at the *GAL1* locus, *GAL1/URA3*, and heterozygous on the Chr5L, *BFP-HIS1*/*GFP*-*ARG4*. Parallel cultures (2× three 1 mL cultures) were generated to assess LOH frequencies of Chr5L and Chr1L. LOH frequency of Chr5L was evaluated using the same protocol as the FACS fluctuation assay previously described, with a total of 48 FACS profiles. The same parallel cultures were also used for counter selection of CFUs having lost either *GAL1* or *URA3* on media containing 2-deoxygalactose (2-DG) or 5-fluoroorotic acid (5-FOA), respectively. The 5-FOA-resistant (5-FOA^R^) CFUs are indicative of individuals that have undergone LOH and have lost the *URA3* marker while 2-DG resistant (2-DG^R^) CFUs are indicative of individuals that have lost the *GAL1* gene. LOH frequency at this locus on Chr1L is calculated by the tabulation of 5-FOA^R^ and 2-DG^R^ CFUs.

## 3. Results

**Figure 2:**
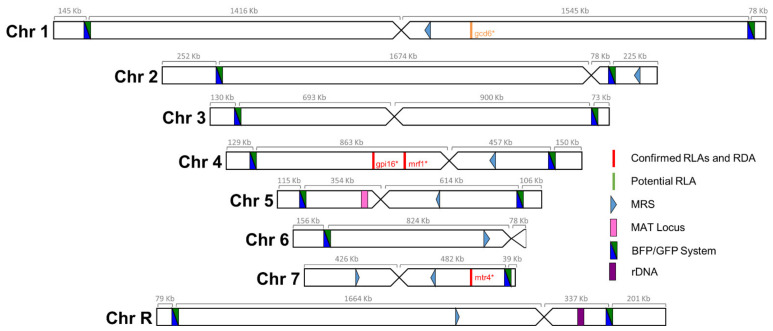
Blue-Green collection. Positions of the BFP/GFP system insertions utilized to measure LOH frequencies. Distances, in kb, between the telomere and the LOH reporter system and the centromere is indicated above each chromosome. Additionally, illustrated are the locations of known recessive lethal alleles (RLA) and recessive deleterious alleles (RDA), major repeat sequences (MRS), mating-type like (MTL) locus, and ribosomal DNA (rDNA) locus.

We sought to build a collection of isogenic strains, each one possessing the BFP/GFP LOH reporter system at a distinct genomic locus, as well as a unique barcode sequence, allowing us to study LOH events throughout the genome of *C. albicans* and permitting pool experiments. The 14 *C. albicans* strains constituting the Blue-Green (BG) collection were selected amongst the 57 strains studied in Marton et al. [35] as they did not display any obvious transformation-induced GCR nor phenotypic aberrations. Our strategy relied on the integration of the BFP/GFP LOH reporter system in the most telomere proximal ≥5 kb intergenic region at both ends of each chromosome (Figure 2), to maximize the recovery of long-tract LOH events occurring on each chromosomal arm. The right arm of the asymmetrical Chr6 was not included in our collection as no intergenic region of ≥5 kb could be identified on this short chromosomal arm. Additionally, the left arm of Chr7, which is highly homozygous in SC5314, was also not included in our analysis as we were unsuccessful in constructing a GCR-free strain carrying the BFP/GFP LOH reporter system on this chromosomal arm.

All strains were derived from the *C. albicans* laboratory reference strain SN148 (His^−^ Arg^−^ Ura^−^ Leu^−^) [34]. This strain was sequentially transformed to integrate (i) the BFP/GFP LOH reporter system, (ii) a unique barcode sequence, and (iii) the leucine marker rendering the strains prototrophic. All engineered strains underwent basic phenotyping to assess if the transformation process drastically impacted their biology, as described in Marton et al. [35]. These strains were shown to form round colonies on both YPD and SD media and to possess comparable doubling times (Figure S1). Additionally, the functionality of the BFP/GFP LOH reporter system was evaluated by challenging the cells with genotoxic treatments such as MMS (DNA alkylating agent) or heat shock (51 °C for 90 s) and assessing fold changes in LOH frequencies. We observed, on average, a nine-fold increase in the frequency of mono-fluorescent cells upon exposure of the BG collection to stress (MMS or heat shock) (Table S1). With all strains of the BG collection displaying an augmentation in the total number of mono-fluorescent cells upon genotoxic stress, the BFP/GFP LOH reporter systems in these 14 strains were defined as being functional. Whole-genome sequencing data permitted us to conclude that these strains did not acquire any large GCR during the strain construction process [35].

### 3.1. A Robust Pipeline to Accurately Evaluate Basal LOH

Fluctuation tests were performed to accurately assess LOH frequency across our collection in various growth conditions. Optimizing our set up, we initially observed high variations in terms of LOH frequency between independent cultures of a given strain in a specific condition. After validating the fact that multiple cytometry analyses from a single culture were very consistent in terms of mono-fluorescent cells frequency, we compared LOH frequency variations between multiple cultures originating from independent colonies of a given strain and between replicate cultures from a single colony preculture (parallel cultures) and saw no differences. Based on these observations, we set up an analysis matrix of LOH frequencies consisting of 24 biological replicates (24 parallel cultures from a single colony preculture), from which 10^6^ cells were analyzed eight times by cytometry (technical replicates), screening a total number of 192 × 10^6^ cells in order to accurately evaluate a median LOH frequency for a given strain in a specific condition, allowing statistical analyses (Figure 1, see material and methods section).

### 3.2. A Genome-Wide Evaluation of Basal LOH Frequency

**Figure 3:**
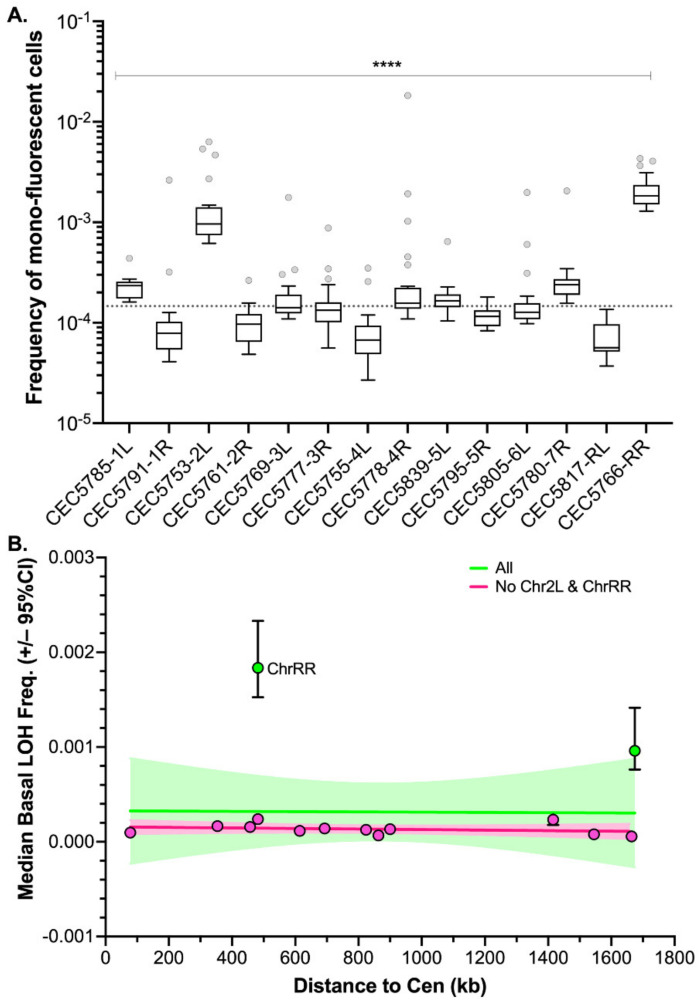
Median basal LOH frequencies across 14 *C. albicans* genomic loci. (**A**) Box plot and whiskers (Tukey) of median basal LOH frequencies obtained upon fluctuation analysis using the Blue-Green collection (*n* = 24 parallel cultures). The median basal LOH frequency of the 14 genomic loci is represented by the gray dashed line. Significant differences between medians were assessed using the Kruskal–Wallis test (**** *p* < 0.0001). (**B**) Relationship between median basal LOH frequency (error: 95% CI) and the distance between the BFP/GFP LOH reporter system and the centromere (CEN) (kb). Linear regression of all 14 loci represented in green (Pearson’s R = −0.0149, *p* = 0.962114) and of 13 loci (without ChrRR possession rDNA locus) represented in pink (Pearson’s R = 0.3826, *p* = 0.19696).

Using the pipeline described above, basal LOH frequencies were evaluated from 24 parallel cultures per strain in YPD at 30 °C; therefore, monitoring 14 distinct genomic loci of the *C. albicans* genome (Figure 3A). As shown in Figure 3A, the median basal LOH frequencies of different genomic loci appeared to be significantly different (Kruskal–Wallis test, *p* < 0.0001). The most elevated LOH frequencies were obtained for the right arm of ChrR (possessing the ribosomal DNA (rDNA) locus) and the left arm of Chr2, the chromosomal arm with the longest LOH screening distance (distance between the BFP/GFP LOH reporter system and the centromere). However, the estimated LOH frequencies, even when excluding the two outliers ChrRR and Chr2L, did not appear to be positively correlated with the distance between the BFP/GFP LOH reporter system and the centromere (Peason’s R = 0.0581, *p*-value = 0.45) (Figure 3B). Overall, basal LOH frequencies remain low throughout the genome, with a median frequency of 1.48 × 10^−5^ LOH.

### 3.3. LOH Frequency Is Influenced by Genetic Background

**Figure 4:**
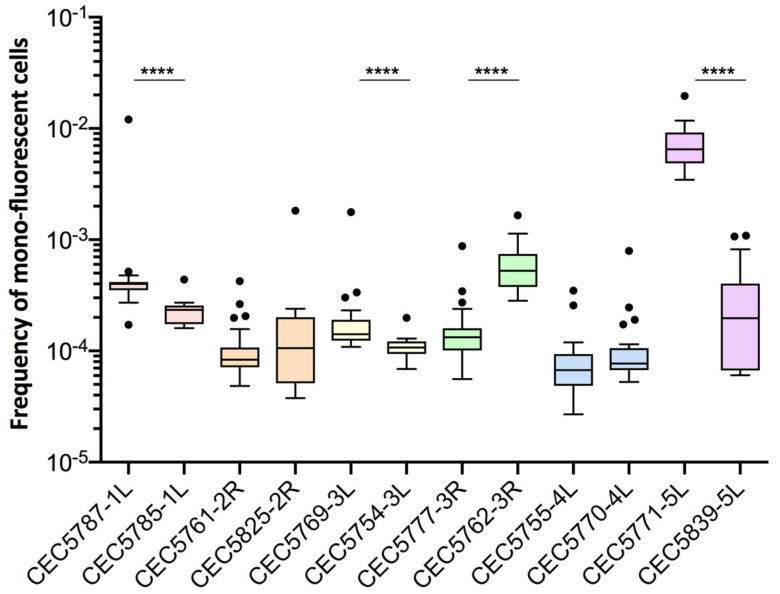
Basal LOH frequencies of two independently constructed strains. Frequency of mono-fluorescence and LOH frequency in YPD represented by box plot and whiskers (Tukey) were assessed by fluctuation analysis (*n* = 24). Frequencies of independently constructed strains were compared using the Mann–Whitney test, *p* < 0.05 (**** *p* < 0.0001).

For certain genomic loci, we were able to retrieve two independently constructed strains and compare LOH frequencies in those pairs. With regards to basal LOH frequencies, 2/6 pairs (Chr2R and Chr4L) showed non-significant differences in terms of LOH frequency (Mann–Whitney test, *p* > 0.05). However, the other four pairs of strains, Chr1L, Chr3L, Chr3R, and Chr5L, exhibited significantly different basal LOH frequencies (Mann–Whitney test, *p* < 0.0001) between independently constructed strains (Figure 4), despite the absence of obvious phenotypic and genomic differences.

**Figure 5:**
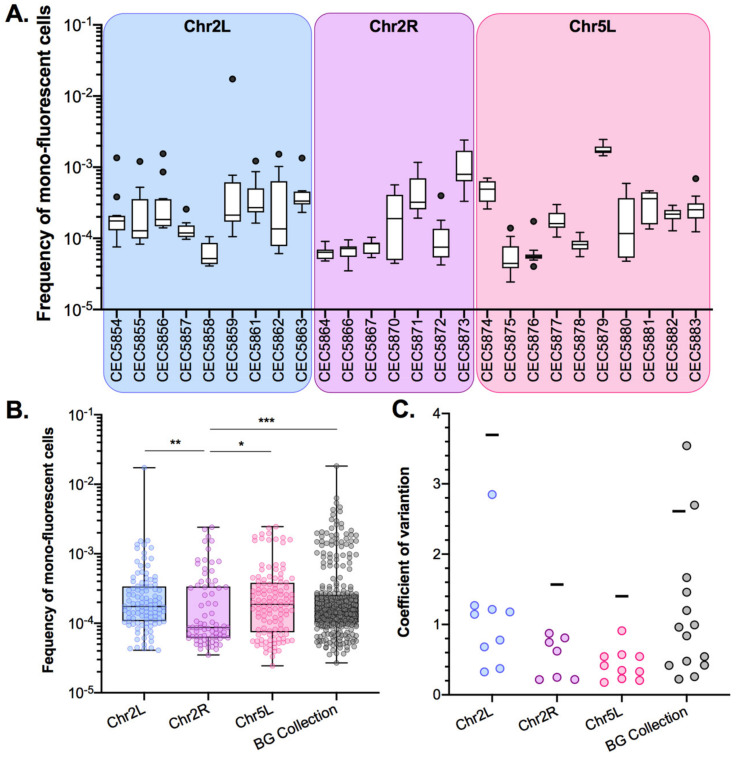
Inter-strain heterogeneity of basal LOH frequency. (**A**). Median frequencies of mono-fluorescence in YPD, basal LOH frequencies, represented as box plots and whiskers (Tukey) were assessed by fluctuation analysis (*n* = 12) for genomic loci: Chr2L (9 strains), Chr2R (7 strains), and Chr5L (10 strains). (**B**). Pooled frequency of mono-fluorescence for the three loci illustrated in the top panel (Chr2L, Chr2R, and Chr5L) and for the 14 strains and loci in the Blue-Green (BG) collection (Figure 2). Overall median frequencies were compared using the Mann–Whitney test, significant differences are shown on the graph * *p* < 0.05, ** *p* < 0.001 and *** *p* < 0.0001). (**C**). Variation of LOH frequencies is represented by the coefficient of variation, where circles represent a given strain while horizontal lines depict the variation between transformants.

These observations suggested that smaller genetic differences, potentially acquired in the process of cell transformation, could influence LOH frequency. As we know that the transformation process of *C. albicans* cells is highly mutagenic [35], we tested our hypothesis by rigorously evaluating heterogeneity in terms of LOH frequency between multiple independently constructed strains. We chose to construct multiple independent SN95-derivatives carrying the BFP/GFP LOH reporter system at three distinct genomic loci, (i) Chr5L (10 strains) because the highest variation was observed for CEC5771-5L, (ii) Chr2L (9 strains), and (iii) Chr2R (7 strains), a chromosome for which LOH frequency increase was seen for one arm (CEC5753-2L) but not the other (CEC5761-2R). All 26 strains carry a functional BFP/GFP LOH reporter system, as illustrated by an average of a 19-fold increase in mono-fluorescent cells upon heat shock or MMS treatment and displayed no obvious phenotypic differences in terms of colony morphology and doubling times (Figure S1, Table S1). Basal LOH frequencies were assessed using the same pipeline as described above, except for conducting 12 parallel cultures rather than 24, thus analyzing a total of 96 × 10^6^ cells by cytometry for each strain. As illustrated in Figure 5A,B, the basal LOH frequencies at three different genomic locations range from 2.44 × 10^−5^ to 1.73 × 10^−2^ and display a high level of heterogeneity in basal LOH frequency between multiple transformants of a single strain (up to 424-, 69-, and 102-fold change for the Chr2L, 2R, and 5L loci, respectively). Figure 5C shows the heterogeneity we observed in terms of LOH frequency, represented by the coefficients of variation, a measure of variability between two different datasets. We observed that the heterogeneity between replicates of a single strain is lower than the heterogeneity observed between independent transformants (Figure 5C) and that the heterogeneity we see at the three tested genomic loci is comparable to the heterogeneity observed when comparing the 14 genomic loci within the BG collection. Our observations led us to conclude that, apart from ChrRR, for which increased basal LOH frequency might be explained by the rDNA locus, the variability we see in YPD between the other loci is comparable to the intrinsic variability we see between multiple independent transformants; although slight but significant differences in median basal LOH frequencies can be seen between different genomic loci when taking into account multiple strains constructed for a given locus (Figure 5B—Kruskal–Wallis test, *p* = 0.011). This is the case for Chr2R and Chr2L (*p* = 0.001) and Chr2R and Chr5L (*p* = 0.0452), for which significant differences (Mann–Whitney test, *n* = 84–120) are obtained (Figure 5B).

### 3.4. Stress Induces Different Responses in Terms of LOH Frequency

**Figure 6:**
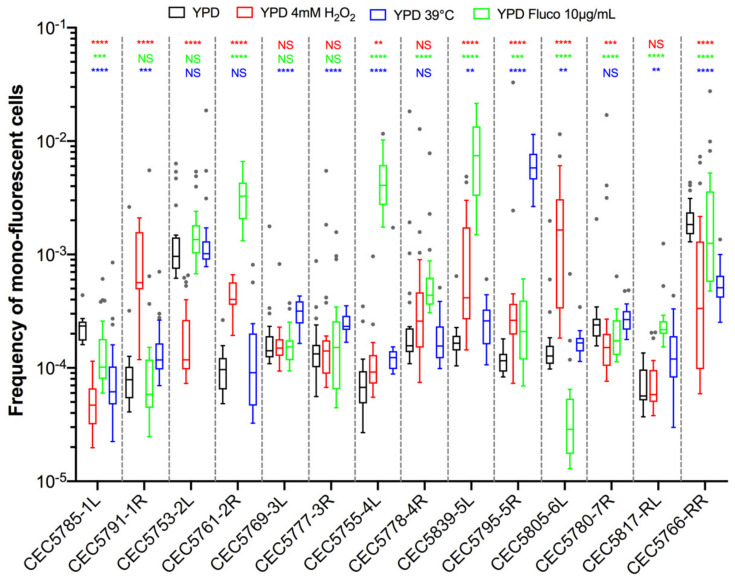
LOH frequency of Blue-Green collection in growth conditions mimicking physiological stresses. Median frequencies of mono-fluorescence, LOH frequency, in YPD (black), 4 mM H_2_O_2_ (red), 10 µg/mL fluconazole (green), and growth at 39 °C (blue) represented as box plots and whiskers (Tukey). LOH frequencies were assessed by fluctuation analysis (*n* = 24) for 14 strains representing fourteen genomic loci (14 strains). Differences of median LOH frequencies, between control condition (YPD) and each stress condition were evaluated using the Mann–Whitney test (NS *p* > 0.05, ** *p* < 0.01, *** *p* < 0.001, and **** *p* < 0.0001).

As stress is known to increase LOH frequency in *C. albicans*, we sought to investigate LOH frequency upon physiologically relevant stresses. Using the same pipeline as described above, we evaluated LOH frequencies in the 14 *C. albicans* strains of the BG collection upon exposure to oxidative stress (4 mM H_2_O_2_), antifungal agents (10 µg/mL fluconazole), and heat (39 °C) (Figure 6). Three different outcomes with regards to LOH frequency were observed upon stress exposure: (i) no effect, (ii) augmentation, or (iii) reduction in LOH frequency, as compared to the basal LOH frequency assessed in YPD.

**Figure 7:**
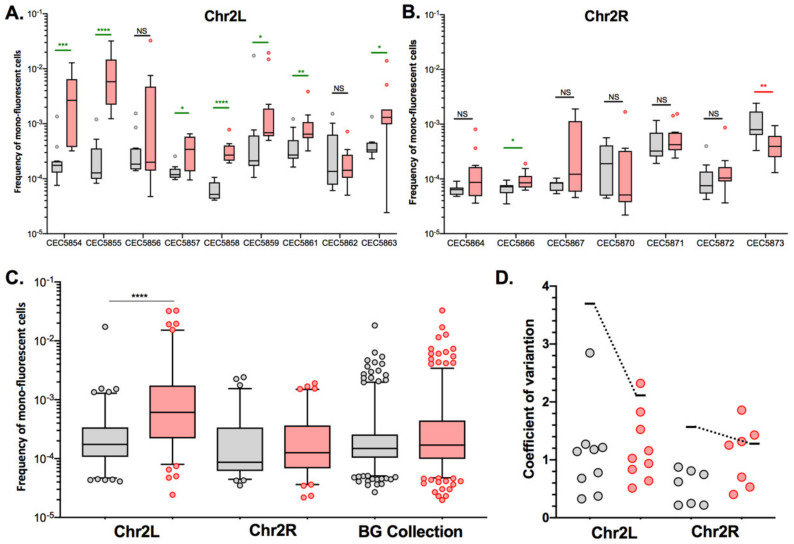
Effect of hydrogen peroxide on LOH frequency in *C. albicans*. Median frequencies of mono-fluorescence, LOH frequency, in YPD (gray), and 4 mM H_2_O_2_ (red) represented as box plots and whiskers (Tukey). LOH frequencies were assessed by fluctuation analysis (*n* = 12). Differences of median LOH frequencies, between control condition (YPD) and the oxidative stress condition were evaluated using the Mann–Whitney test (NS *p* > 0.05, * *p* < 0.05, ** *p* < 0.01, *** *p* < 0.001, and **** *p* < 0.0001). Significant augmentation and reductions of LOH frequencies are identified in green and red, respectively, for panels (**A**,**B**). (**A**) Representation of median LOH frequencies for 9 strains possessing the BFP/GFP LOH reporter system on Chr2L. (**B**) Representation of median LOH frequencies for 7 strains possessing the BFP/GFP LOH reporter system on Chr2R. (**C**) Heterogeneity and median LOH frequency of Chr2L (9 strains), Chr2R (7 strains), and Blue-Green (BG) collection (14 strains) by pooling of multiple strains. (**D**) Heterogeneity of LOH frequencies represented by coefficients of variation. Each circle represents the coefficient of variation for a single strain while the variation between strains is shown by a horizontal line.

Because of the basal LOH frequency heterogeneity described above, we questioned if the H_2_O_2_-induced changes in LOH frequencies we observed for certain chromosomes could be interpreted as a true stress-induced chromosome-specific instability or could just illustrate the natural variability we observe between multiple transformants of the same strain. As shown in Figure 6, strains CEC5753-2L and CEC5761-2R display opposite responses in terms of LOH frequency in response to H_2_O_2_ treatment, with an eight-fold decrease for CEC5753-2L and a four-fold increase in LOH frequency for CEC5761-2R. To test if these outcomes could be reproduced with multiple independent strains for the given loci, we used the nine Chr2L and seven Chr2R independently constructed strains, as described above, to assess LOH frequencies upon exposure to hydrogen peroxide (4 mM). Here again, the independently constructed strains display different responses to H_2_O_2_ in terms of genome stability (Figure 7A,B). In contrast to the conclusions drawn from the LOH analysis in single strains within the BG collection (Figure 6), the analysis performed with multiple transformants for a given locus allows us to conclude that only Chr2L exhibits an augmentation of median LOH frequency upon H_2_O_2_ exposure (Figure 7C), while nonsignificant LOH frequencies were assessed for Chr2R and the BG collection as a whole. Interestingly, while the variability between replicates for a specific strain is logically less in YPD than in response to H_2_O_2_ treatment, the opposite is true concerning the variability between multiple independent transformants (Figure 7D). The overall specific impact of the genotoxic stress seems to “mask” the intrinsic variability due to genetic background differences between multiple independent transformants seen in YPD.

### 3.5. Stress May Not Lead to a General Response Triggering Overall Genome Instability

**Figure 8:**
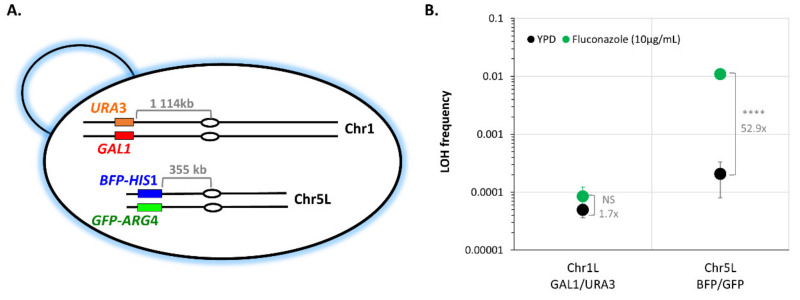
Assessing LOH frequency of two distinct *C. albicans* chromosomes within a single strain. (**A**) Illustration of the strain possessing the BFP/GFP LOH reporter system on Chr5L and the GAL1/URA3 system of Chr1L. Centromeres are indicated by ovals and distances between reporter systems and centromeres are indicated in gray. (**B**) Median frequencies of mono-fluorescence, LOH frequency, in YPD (black), and 10 µg/mL fluconazole (green), +/− standard deviation. LOH frequencies were assessed by fluctuation analysis (two independent strains, *n* = 6). Fold changes of median LOH frequencies, between YPD and fluconazole conditions are indicated on the plot in gray and statistical differences were evaluated using *t*-test (NS *p* > 0.05 and **** *p* < 0.0001).

We then sought to understand if the stress-induced LOH increase we observed at the level of a specific chromosome illustrates a genome-wide general response triggering overall genome instability or if, conversely, chromosomes do react independently to a specific stress. As shown in Figure 6, CEC5839-5L shows an increase in LOH frequency upon fluconazole treatment, while CEC5785-1L does not. We took advantage of the GAL1 locus on the left arm of Chr1 to build a strain possessing two different LOH reporter systems: the BFP/GFP LOH reporter system localized on the left arm of Chr5 and the *GAL1*/*URA3* system on the left arm of Chr1 (Figure 8A). In YPD, we obtained median basal LOH frequencies of 4.95 × 10^−5^ and 2.07 × 10^−4^ on Chr1L and Chr5L, respectively (Figure 8B). Upon exposure to fluconazole (10 µg/mL), no changes in LOH frequency on Chr1L were detected (Mann–Whitney test, *p* < 0.142), while LOH frequency on Chr5L shows a statistically significant 52.9-fold increase (*t*-test, *p* < 1.185 × 10^−9^) (Figure 8B), demonstrating that within the same strain, chromosomes will react differently to the same genotoxic stress, with certain loci being more prone to undergo LOH than others. These differences are also observed when normalizing LOH frequencies to the distance between the LOH reporter systems and the centromeres.

## 4. Discussion

Eukaryotic genomes are naturally dynamic [38], under the constant watch of specific surveillance mechanisms required to reach the fine-tuned balance between genomic stability, which ensures the transmission of genetic content to the next generation, and genomic plasticity, which promotes the acquisition of new genetic variants and defines the adaptation potential of microorganisms to a fluctuating environment. A particular form of chromosome instability, namely LOH, is sufficient to provide a selective growth advantage and is often seen in tumors. LOH occurs when a cell that is originally heterozygous at a locus loses one of its two alleles at that locus. This phenomenon is particularly relevant in heterozygous diploid organisms where LOH allows recessive phenotypes to be expressed.

We took advantage of the human fungal pathogen *C. albicans* to investigate the dynamics of genome-wide LOH in a diploid heterozygous eukaryotic genome. By monitoring LOH frequency on each arm of the eight chromosome pairs, our work revealed high variability in terms of genome stability not only at the locus level but also at the strain level, highlighting inter-strain, intra-strain, and inter-chromosome variations, suggesting that multiple stochastic parameters influence genome dynamics in *C. albicans*.

Our initial analysis, looking at a single strain per locus, showed that the median basal LOH frequencies of different genomic loci appeared to be significantly different in the absence of stress (Figure 3A). Two outliers were observed, namely ChrRR (possessing the ribosomal DNA (rDNA) locus) and Chr2L, the chromosomal arm with the longest LOH screening distance (distance between the BFP/GFP LOH reporter system and the centromere) (Figure 3). Instability of the rDNA locus is a known feature of the *C. albicans* genome [39]. This locus encompasses the 18S, 5.8S, 25S, and 5S rRNAs organized as tandem repeating units, whose number can vary from 21 to 176 copies depending on the strains and growth conditions. The variation in copy number, resulting from frequent unequal intrachromosomal recombination, translates into large-scale shifts in the rDNA locus size, ranging from 244 kb to 2200 kb. The recombinogenic properties of the rDNA locus are also illustrated by the fact that the majority of *C. albicans* clinical isolates are homozygous downstream of the rDNA locus and up to the telomere. Regarding Chr2L, it is largely accepted that the probability of a recombination event between a locus and its centromere increases with distance from the centromere [5,33]. While Forche et al. demonstrated that LOH rates at different loci generally correlate with an increased distance from the centromere in *C. albicans* [5], such correlation was not reached in our hands (Figure 3B). This discrepancy can be explained by the high variability revealed by our work, in terms of LOH frequency between independent strains carrying the LOH reporter system at the same locus. Additionally, the localizations of our LOH reporter systems were designed to recover a maximum amount of distal LOH events whereas the study of Forche et al. used randomly distributed loci. Thus, the latter inconsistency between the two studies could also be explained by the absence of correlation between distal LOH frequency and chromosome arm length [40]. Indeed, our analysis, extended to several independent transformants for the same locus, led us to conclude that, apart from ChrRR for which increased basal LOH frequency might be explained by the rDNA locus, the variability we see in YPD between the other loci is comparable to the intrinsic variability we see between multiple independent transformants (Figure 5B); although slight but significant differences in median basal LOH frequencies can be seen between different genomic loci when taking into account multiple strains constructed for a given locus. This is the case for Chr2R, for which we observed a significant lower basal LOH frequency as compared to Chr2L and Chr5L (Figure 5B).

Stress-inducible mutagenesis mechanisms have been described in bacteria, yeast, and human cancer cells. Particularly in hybrid yeast strains, positive selection on existing heterozygosity for the adaptation to a new environment through LOH has been documented [41]. Relying on LOH to generate phenotypic diversity is all the more relevant in a heterozygous diploid microorganism whose mode of reproduction is mainly clonal as it allows *C. albicans* to avoid Haldane’s sieve, with recessive beneficial mutations that can be revealed and can contribute to rapid evolutionary adaptation through LOH [17]. Therefore, we were interested in having a genome-wide picture of LOH dynamics in response to relevant stresses. Although we were able to conclude that Chr2L is more prone to undergo LOH as compared to Chr2R in response to oxidative treatment (Figure 7C), our data especially highlighted the great natural variability that can be observed again between multiple transformants of the same strain (Figure 7D). It was striking to see independent transformants displaying an opposite response in terms of LOH frequency upon exposure to H_2_O_2_, with some showing a decrease while others showed an increase in LOH frequency at a single genomic locus (Figure 7A,B). Remarkably, our results demonstrated that the stress-induced genome instability we observed for a specific chromosome is not the consequence of a general response that triggers overall genome instability. Indeed, we showed that within the same strain, Chr1L and Chr5L will react differently to fluconazole treatment, with Chr5L being more prone to undergo LOH than Chr1L (Figure 8). Altogether, our results revealed a high heterogeneity between multiple transformants in terms of genome stability and highlighted the fact that conclusions drawn from the genome analysis performed on a single strain must be tempered by appropriate cautions. Knowing now that data from two independent transformants can go in opposite directions, only analysis from multiple transformants should be performed to confidently conclude on a trend in terms of LOH frequency at a specific locus.

This elevated variability that seems to be omnipresent in *C. albicans* can be seen at various levels. Of course, by the very definition of a fluctuation analysis, variations in terms of LOH frequency between parallel cultures from a unique pre-culture were anticipated based on the timing of the appearance of the new mutation in a single cell during growth of the population. In addition, inter-chromosome variability in terms of genome stability in response to stress was already suggested by Forche et al. [5] when they showed that fold changes for LOH rates in response to heat, oxidative stress, and antifungals were highly variable between strains with an LOH reporter system at five locations in the genome. Inter-strain variability in additional aspects of *C. albicans* biology were also suggested in other studies such as the work by Huang et al. [29] on differences in the regulation of biofilm formation in five clinical isolates. Altogether, other genomic features or other parameters seem to influence LOH occurrence in *C. albicans*. These parameters are not yet fully comprehended but several scenarios can be considered. (i) We know that the process of transformation in *C. albicans* is highly mutagenic with 33% to 60% of transformed strains displaying GCRs, depending on the protocol [35]. It would be interesting to perform deep whole-genome sequencing of supposedly isogenic-independent transformants to see if the phenotypic variations observed in terms of LOH frequency is a fair representation of transformation-induced genomic differences. (ii) Cells could also differ in terms of the level of gene expression as it has been reported in *S. cerevisiae*, in particular expression noise of genes affecting HR activity and responsible for cell-to-cell heterogeneity in terms of HR rate [42], which could thus impact the LOH frequency. (iii) Variations in epigenetic states between strains could also explain the LOH frequency heterogeneity as it has been shown that chromatin structure influences the occurrence of recombination events (reviewed in [43]). Therefore, the comparison of methylation profiles between strains could shine light on the causes of LOH frequency heterogeneity. (iv) It has been shown in cancer genomes that LOH preferentially occurs in early replicating regions, where interference between replication and transcription machineries can lead to the formation of double-strand breaks [44]. Based on the observations made by Koren et al. [45] that DNA replication timing is shaped by genetic polymorphisms in human cells, it would be interesting to investigate whether variations in terms of the DNA replication origins landscape between different strains could explain the variations in LOH frequency. (v) Studies on the relative frequency of LOH events between human chromosomes revealed that the relative frequency of LOH events per chromosome had significantly inverse correlations with the distance between homologous chromosomes in the nucleus [44]. Eukaryotic chromosomes occupy distinct nuclear territories, such that some pairs of homologous chromosomes are closer to each other than other pairs. Little has been done regarding chromosome organization in the *C. albicans* nucleus. Variations from cell to cell could impact the evaluation of LOH frequency in a given *C. albicans* population.

Using molecular approaches to understand *C. albicans* biology, we tend to see phenotypic variability as a barrier that prevents us from carrying out conclusive statistical analysis when we should in fact embrace it as a fair representation of the potential of adaptation of this human fungal pathogen and a functional consequence of genetic variations. One should remain cautious regarding certain statements that have been made on the action of certain genetic and environmental alterations on the genome dynamics of *C. albicans*.

## Data Availability

Not applicable.

## Supplementary Materials

The following supporting information can be downloaded at: https://www.mdpi.com/article/10.3390/jof8070650/s1, Figure S1: Doubling time of *C. albicans* strains used within this study.; Table S1: Fold changes of monofluorescent cells upon stress exposure permitting to validate the functionality of the BFP/GFP LOH reporter systems within *C. albicans* strains used in this study.; Table S2: List of strains and plasmids used throughout this study.; Table S3: Primers used throughout this study.
