# Supplementary figures and images for: Multiple Stochastic Parameters Influence Genome Dynamics in a Heterozygous Diploid Eukaryotic Model
Source: J Fungi (Basel). 2022 Jun 21;8(7):650. doi: 10.3390/jof8070650 (PMC9323731; doi:10.3390/jof8070650)

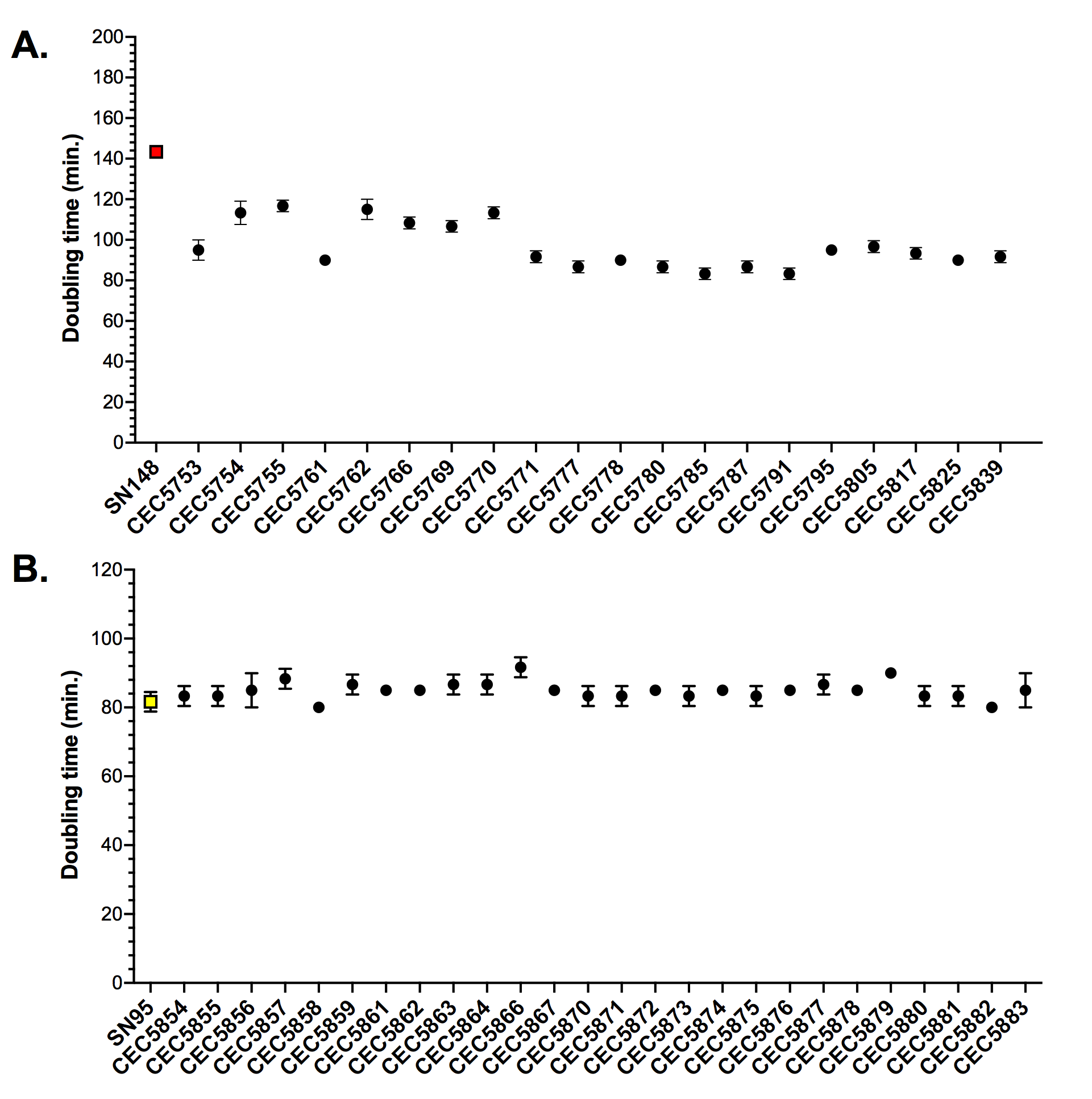

Supplement: Supplementary file 1 [file jof-08-00650-s001.zip › Supplementary_Materials/FigureS1_Marton_et_al_PlosBiology.tiff]
